# Human cardiac tissues produce lower contractile stress and exhibit slower cross-bridge cycling in type 2 diabetes

**DOI:** 10.1186/s12933-025-02820-7

**Published:** 2025-07-03

**Authors:** J. H. Musgrave, J.-C. Han, M.-L. Ward, N. Kang, A. J. Taberner, K. Tran

**Affiliations:** 1 Auckland Bioengineering Institute, University of Auckland, Auckland, New Zealand; 2 Department of Physiology, University of Auckland, Auckland, New Zealand; 3 Greenlane Cardiothoracic Surgical Unit, Auckland City Hospital, Auckland, New Zealand; 4 Department of Engineering Science and Biomedical Engineering, University of Auckland, Auckland, New Zealand

## Abstract

**Background:**

Diabetes mellitus elevates the risk of developing heart failure and increases associated mortality rates. While the clinical features of diabetic cardiomyopathy have been extensively studied, the effects of diabetes and associated changes in metabolic state on contractile cross-bridge function are less well understood. Using our suite of experimental methods designed to measure cross-bridge kinetics and metabolite sensitivity, we aim to elucidate the mechanistic pathways by which cross-bridge alterations contribute to myocardial dysfunction observed in diabetic cardiomyopathy.

**Methods:**

Atrial trabeculae from non-diabetic and type 2 diabetic patients without heart failure were permeabilised and subjected to a series of experiments to measure their cross-bridge function and sensitivity to metabolites. Muscle active stress production and muscle active complex modulus measurements were gathered across different concentrations of ATP and inorganic phosphate (Pi) for the two groups of muscles. To link these functional data to tissue structural alterations, confocal imaging was performed to quantify the trabecula myofilament content and SWATH-MS was performed to measure the composition of myosin isoforms.

**Results:**

Diabetic trabeculae generated 20% lower active stress and had 16% lower cross-bridge stiffness on average. The reduction in active stress production can be attributed to a lower density of myocytes in the diabetic muscles. The diabetic trabeculae also had a 24% reduction in characteristic frequencies, reflecting slower cross-bridge cycling kinetics. This result was consistent with the measurement of a reduced fraction of the alpha myosin isoform in this group of patients. The interaction between diabetic status and metabolites was more complex. Although we found that diabetes did not affect the force response to changes in ATP or Pi concentrations, we found that the stiffness of cross-bridges had a lower sensitivity to ATP in diabetic tissues.

**Conclusions:**

Our key results point to potential mechanisms of clinical dysfunction in diabetic heart tissue. Lower active force production in diabetic trabeculae suggests that these patients are developing contractile dysfunction. Furthermore, slower cross-bridges can contribute to diastolic dysfunction, especially at higher heart rates, by prolonging cardiac relaxation.

**Graphical Abstract:**

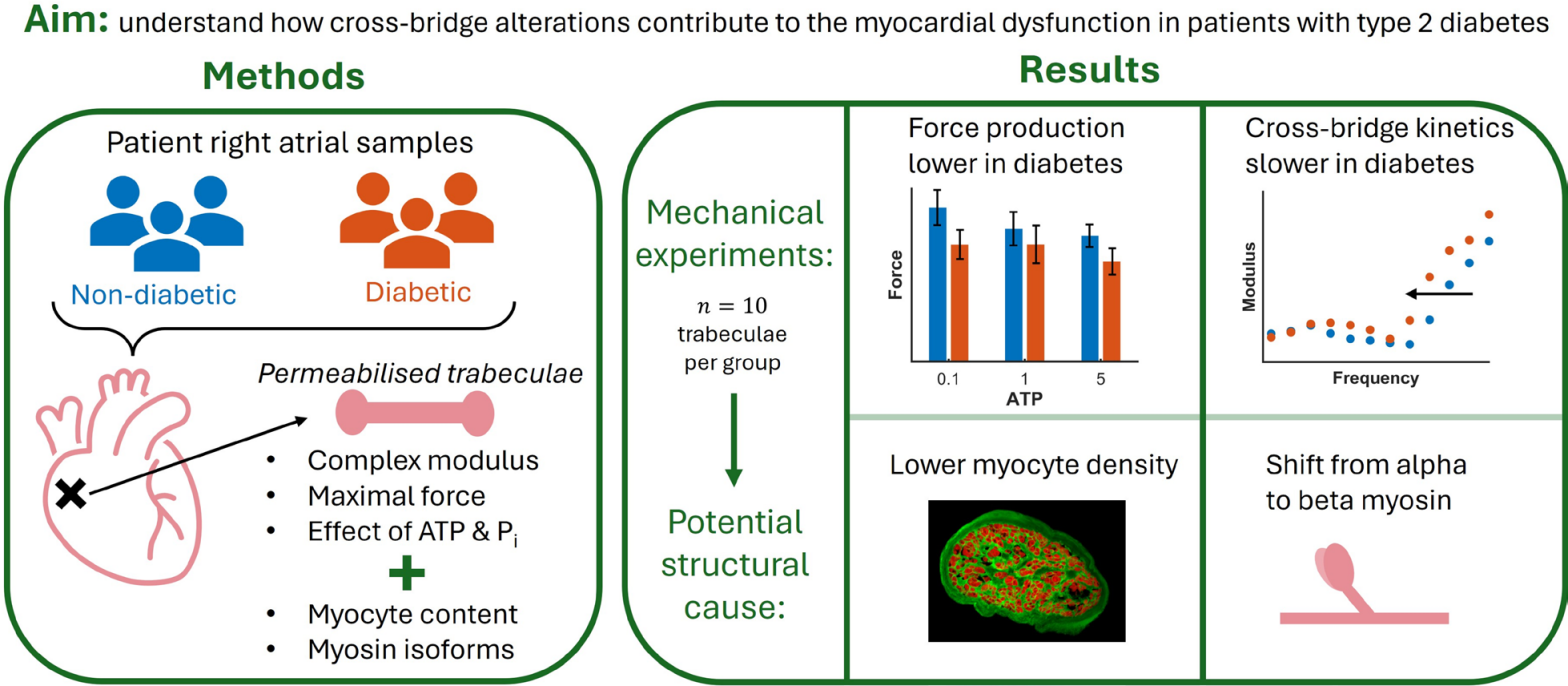

## Introduction

With diabetes and heart failure both being among the leading causes of death globally [1], improving our understanding of these complex diseases and their interaction is paramount. Patients with type 2 diabetes have more than twice the risk of developing cardiovascular disease [2] and there is a four-fold higher mortality risk among those who develop heart failure [3]. The effects of diabetes on the heart occur across mechanical and energetic domains. Due to the metabolic nature of the disease, cardiac mitochondrial dysfunction is often present in diabetic patients, linked to a shift in primary fuel substrates and consequently reduced energy efficiency [4]. Metabolic impairments also result in altered concentrations of energy molecules in the heart, specifically lower availability of adenosine triphosphate (ATP) [5] and an increase in inorganic phosphate (P_i_), a key product of ATP hydrolysis [6]. From a mechanical standpoint, diabetes is linked to organ-level systolic dysfunction [3], where the heart does not produce sufficient pressure when contracting; and diastolic dysfunction [2], impairment in the heart’s ability to relax between beats.

While many interacting systems contribute to cardiac function and dysfunction, the essential contractile behaviour of the heart is driven by cellular cross-bridge activity. The pressure generation and ejection phases of the cardiac cycle are driven by a cyclical interaction of actin and myosin filaments within cardiomyocytes, which is in turn driven by ATP hydrolysis. Following electrical stimulation and excitation-contraction coupling, cross-bridge cycling generates tissue contraction, while the unbinding phases of this cycle also contribute to the rate of subsequent relaxation [7]. Contractile function in cardiac tissues also depends on the concentration of cytosolic metabolites, both in terms of the free energy available for ATP hydrolysis and the allosteric interaction between metabolites and cross-bridge cycling kinetics [8]. Alterations in cardiac contractile function in diabetes, therefore, arise from complex interactions between the mechanical and energetic domains.

Despite cross-bridge cycling being a key driver of cardiac function, there is currently limited experimental data characterising how diabetes affects cross-bridge function and its sensitivity to cellular metabolites. Given the recognised value of multiscale computational models in furthering basic science research and in the growing field of personalised medicine, a dataset suitable for parameterising a model representing this system is of particular interest.

We have previously developed experimental methods involving measurement of the complex modulus [9] that are well-suited to addressing this gap in the experimental literature. The complex modulus represents the viscoelastic properties of contracting muscle, and reflects the interacting rates of the biochemical cross-bridge cycling processes as well as their fundamental mechanical properties [10]. These experimental methods produce a dataset which describes the cross-bridge properties and their sensitivity to metabolites, and is sufficiently rich for parameterising biophysical cross-bridge models.

In this study, we used these methods to investigate cellular-level changes in mechanics and metabolite sensitivity in human tissue samples from patients with type 2 diabetes. As the samples came from patients who have not yet developed heart failure, this allowed for study of the earlier stages of naturally-acquired diabetic heart disease. The findings from our functional experimental methods are complemented by confocal imaging of tissue myocyte content and mass spectrometry (SWATH-MS) analysis of contractile proteins which provide an in-depth investigation of the effect of diabetes on human cardiac tissue function.

## Methods

### Study information

Human cardiac tissue samples used in this study were collected from Auckland City Hospital. Patients consented to the study prior to undergoing coronary artery bypass grafting (CABG) surgery, as per Human and Disability Ethics Committee of New Zealand approval (HDEC PR6432).

Trabeculae were collected from eight patients with type 2 diabetes ($n=10$) and from seven non-diabetic patients ($n=10$). Up to two trabeculae were dissected from the same patient tissue sample. There were three female patients in the diabetic group and one in the non-diabetic group. The patient groups did not differ in age, Body Mass Index (BMI) and estimated pre-operative ejection fraction (Table 1). E/e′ ratio and HbA1c were significantly higher in the diabetic group. None of the patients had heart failure at the time of surgery.

**Table 1:** Key patient data associated with the trabeculae measured in the study

	Non-diabetic $n=7$	Diabetic $n=8$	*p*-value
Gender (Male/Female)	6M/1F	5M/3F	–
Age (years)	64.9 ± 3.1	64.9 ± 3.4	0.39
BMI (kg $\hbox {m}^{-2}$)	27.6 ± 1.0	31.8 ± 1.7	0.07
Ejection fraction (%)	46.7 ± 3.5	50.3 ± 4.8	0.75
E/e’ ratio	7.36 ± 0.64	11.57 ± 1.22	0.030.03*
HbA1c (mmol/mol)	38.4 ± 1.0	58.5 ± 4.8	<0.01*

* $p< 0.05$, indicating statistical significance by t-test. Note that HbA1c was reported only for five of the seven non-diabetic patients and E/e’ was not reported for three patients (two non-diabetic, one diabetic)

### Sample procurement

A small section of tissue from the right atrial appendage (RAA) was removed during the surgery, with maximum dimensions of 5 mm $$\times $$ 5 mm $$\times $$ 20 mm. Immediately after excision, the sample was transferred into a chilled Krebs-Henseleit (KH) buffer and rapidly (under 10 min) brought back to the laboratory for dissection. The KH buffer used for transport and dissection was made up as follows: 118 mM NaCl, 4.75 mM KCl, 1.18 mM $$\hbox {MgSO}_4$$, 1.18 mM $$\hbox {KH}_2$$$$\hbox {PO}_4$$, 24.8 mM $$\hbox {NaHCO}_3$$, 11 mM glucose, 25 mM BDM, 0.25 mM $$\hbox {CaCl}_2$$ and continuously bubbled with 95 % $$\hbox {O}_2$$/5 % $$\hbox {CO}_2$$ to maintain pH of 7.4.

Upon arrival at the laboratory, the sample was pinned in place to a dissection bath and superfused with continuous flow of KH buffer at room temperature. Free-running trabeculae were dissected from the endocardial surface of the atrial sample and placed into a 4 °C permeabilisation solution for approximately 20 h. The permeabilisation solution had a pH of 7.0, an ionic strength of 180 mM and contained 7 mM EGTA, 20 mM imidazole, 4 mM MgATP, 1 mM Mg^2+^, 10 μg/mL leupeptin, 30 mM BDM, 1 % v/v Triton, 50 % v/v glycerol.

Following trabecula dissections, subsamples spanning the wall thickness of the patient samples were dissected in blocks, flash-frozen in liquid nitrogen and stored at − 80 °C for 9–18 months.

### Mechanical measurements

Our custom experimental device [11, 12] was used to perform a series of mechanical experiments on the permeablised trabeculae. These experiments captured active and passive properties of the muscle, as well as the response of the muscle to changes in ATP and P_i_ concentration.

Relaxing, pre-activating and activating solutions were prepared for the experiments (summarised in Table 2). All of the experimental solutions were adjusted with KCl to produce a final ionic strength of 180 mM and with KOH to produce a pH of 7.0 at the experimental temperature (37 °C). The set-up phase of the experiments was performed at room temperature, so relaxing solution was also prepared at 22 °C. The final concentrations of each metal, ligand, and metal-ligand complex were calculated using version 2.5 of the MaxChelator computer program [13].

**Table 2:** Solutions used for contractile experiments

Compound	Relaxing	Pre-activating	Activating (baseline)
EGTA (mM)	7	0.5	7
HDTA (mM)	–	6.5	–
Imidazole (mM)	20	20	20
CrP (mM)	14.5	14.5	14.5
MgATP (mM)	5	5	5
P_i_ (mM)	1	1	1
Free Mg^2+^ (mM)	1	1	1
Creatine Kinase (U/mL)	–	–	240
pCa	9	9	4.5
Temperature (°C)	22/37	37	37

All solutions had a pH of 7.0 at the stated temperature

Following permeabilisation, each trabecula was rinsed with relaxing solution and mounted into the experimental device at room temperature. Muscles were stretched to a sarcomere length of 2.2 μm (optimal length) where their final experimental lengths were measured. Muscle force was set to be zero at 85 % of the optimal muscle length (corresponding to a sarcomere length of 1.87 μm). After the set-up phase was complete, the experimental baths were heated to 37 °C.

In the subsequent mechanical interventions, complex modulus measurements were performed at 12 logarithmically-spaced frequencies between 0.18 Hz and 100 Hz. At each frequency, the force generated by the muscle was measured under sinusoidal length perturbations, with a peak-to-peak amplitude of 0.25 % of muscle length. The complex modulus was calculated at each frequency, by dividing the fast Fourier transform of force by the fast Fourier transform of normalised muscle length. Measurements taken in relaxing solution (pCa 9.0) are referred to as passive measurements, while those taken in activating solution (pCa 4.5) are referred to as total measurements, where total = passive + active. Active measurements, which reflect the cross-bridge properties alone, were thus found as the difference between equivalent passive and total measurements.

#### Initial mechanics protocol

The first stage of experiments, where the passive and baseline active muscle properties were measured, was identical across all muscles. Prior to activation, the muscle was switched into a bath of pre-activating solution for 1 min. Under baseline activating solution (5 mM ATP and 1 mM P_i_), the muscle was held at a constant length until a steady-state force was reached. Here, we measured the total complex modulus (as previously described) as well as the total force-length relationship. The steady-state forces were recorded after shortening the muscle by 5%, 10% and 15%, as well as at the optimal muscle length. Following these experiments, the muscle was switched back into relaxing solution and passive measurements were collected. The passive complex modulus was measured at optimal length and the steady-state passive force was recorded at 100%, 95%, 90% and 85% of optimal muscle length.

#### Metabolite-sensitive mechanics protocol

Following the initial protocol, a series of experiments were performed to assess the influence of ATP and P_i_ concentrations on the mechanics of the muscle. Five different conditions of metabolite concentrations were used (Table 3), resulting in three different concentrations for each of ATP and P_i_.

**Table 3:** ATP and P_i_ concentrations of the five activating solutions used for this study

Condition	[MgATP] (mM)	[P_i_] (mM)
Baseline (B)	5	1
Low ATP (1A)	1	1
Super-low ATP (0.1A)	0.1	1
No P_i_ (0P)	5	0
High P_i_ (10P)	5	10

To account for the effect of order dependence on the results, a balanced Latin square experimental design was used to prescribe the order of presentation of the metabolite solutions. This design included 10 unique permutations of exposure order for the five different metabolite interventions. Within the diabetic and non-diabetic patient groups, each of the exposure patterns was used for one trabecula.

For each activating solution, the muscle was, again, initially placed in pre-activating solution for 1 min. The muscle was then switched to a bath containing one of the five activating solutions listed in Table 3 until the force generated reached a plateau. After reaching a steady state, the total complex modulus of the muscle was measured before it was returned to a relaxing solution in preparation for the next intervention.

Following the second and fifth interventions, the muscle was activated in the baseline solution and the force was recorded to enable consistent measurement of any force rundown or drift throughout the experiment.

### Experimental analysis

All muscle forces and complex moduli were converted to units of stress (Pa) following the experiment. This normalises the force measurements to the cross-sectional area of the trabecula and provides more consistent comparison between muscles. The measurement of cross-sectional area used for this normalisation is detailed in Section “Trabecula structural imaging”.

To account for the unavoidable reduction in maximum force production throughout the experiment (rundown), a linear model was used to adjust the active stress measurements. The slope of force rundown was found by fitting the four measurements of steady-state stress made in baseline activating solution for each trabecula. Using the relative time elapsed in the experiment, this slope was used to adjust all active stresses. The adjustment ensured that each stress measurement was in proportion to that which was recorded in the initial baseline mechanics protocol.

To assess the frequency behaviour of the muscles quantitatively, the active complex moduli under different metabolite conditions were fitted to an exponential model [14] with two processes:

1 $$\begin{aligned} Y_a=H+\frac{Bif}{if+b}+\frac{Cif}{if+c}, \end{aligned}$$

where $Y_a$ is the active complex modulus, *H*, *B* and *C* represent the magnitudes of the components, *b* and *c* are the characteristic frequencies of the two exponential processes, and *f* is the frequency of the complex modulus. This is the simplest model that captures the most important frequency information of the complex modulus, namely the dip (*b*) and high pass (*c*) components. The model was fitted to the active complex modulus of each muscle under all metabolite conditions in MATLAB, using a nonlinear least-squares algorithm.

To assess if there was an effect of diabetes on the measured results, muscles were grouped depending on the diabetic status of the patients, and statistical analyses were performed in RStudio. Two-way ANOVA was used to determine if there was a main effect of diabetes on the dynamic modulus (magnitude of the complex modulus) across all frequencies, the active stress generated across the different metabolite conditions and both active and passive stresses across different muscle lengths. Three-way ANOVA was used to assess the effect of diabetes on the dynamic modulus across all frequencies and across the metabolite conditions. The effect of diabetes on these metrics was deemed significant when $p<0.05$ for the patient group effect. Pairwise comparisons were made using Tukey’s test when the ANOVA group effect was significant and relevant comparisons were reported when significant. Note that for most tests only the overall group effect was significant. The difference in metabolite sensitivity between the patient groups was deemed significant in ANOVA analyses when $p<0.05$ for the interaction effect between patient group and metabolite condition.

All measurements for the non-diabetic and diabetic muscle groups are presented in text and figures as the group mean ± standard error.

### Trabecula structural imaging

After the mechanical experiment, trabeculae were prepared for immunolabelling and imaging. Each trabecula was stretched to its experimental length and fixed with 2 % paraformaldehyde (PFA) in phosphate-buffered solution (PBS) for 10 min at room temperature. Fixed trabeculae were cryoprotected in PBS with 30 % sucrose and then stored in optimal cutting temperature compound at − 80 °C until cryosectioning, where six to eight 10 μm transverse sections were taken at >100 μm intervals across the length of the trabecula.

The antibody labelling protocol to stain the tissue sections was adapted from [15]. Sections were rehydrated with PBS and blocked for 1 h with Image iT FX Signal Enhancer. Wheat Germ Agglutinin (WGA) conjugated with Alexa Fluor 488 was added at a concentration of 1 mg/L and Alexa Fluor 594 phalloidin was added at 0.264 μM, to PBS with 1% bovine serum albumin (BSA) and 0.01 % sodium azide. Prolong Gold antifade reagent was used to mount coverslips on top of the sections. The slides were cured at 4 °C for 72 h before imaging.

**Fig. 1:**
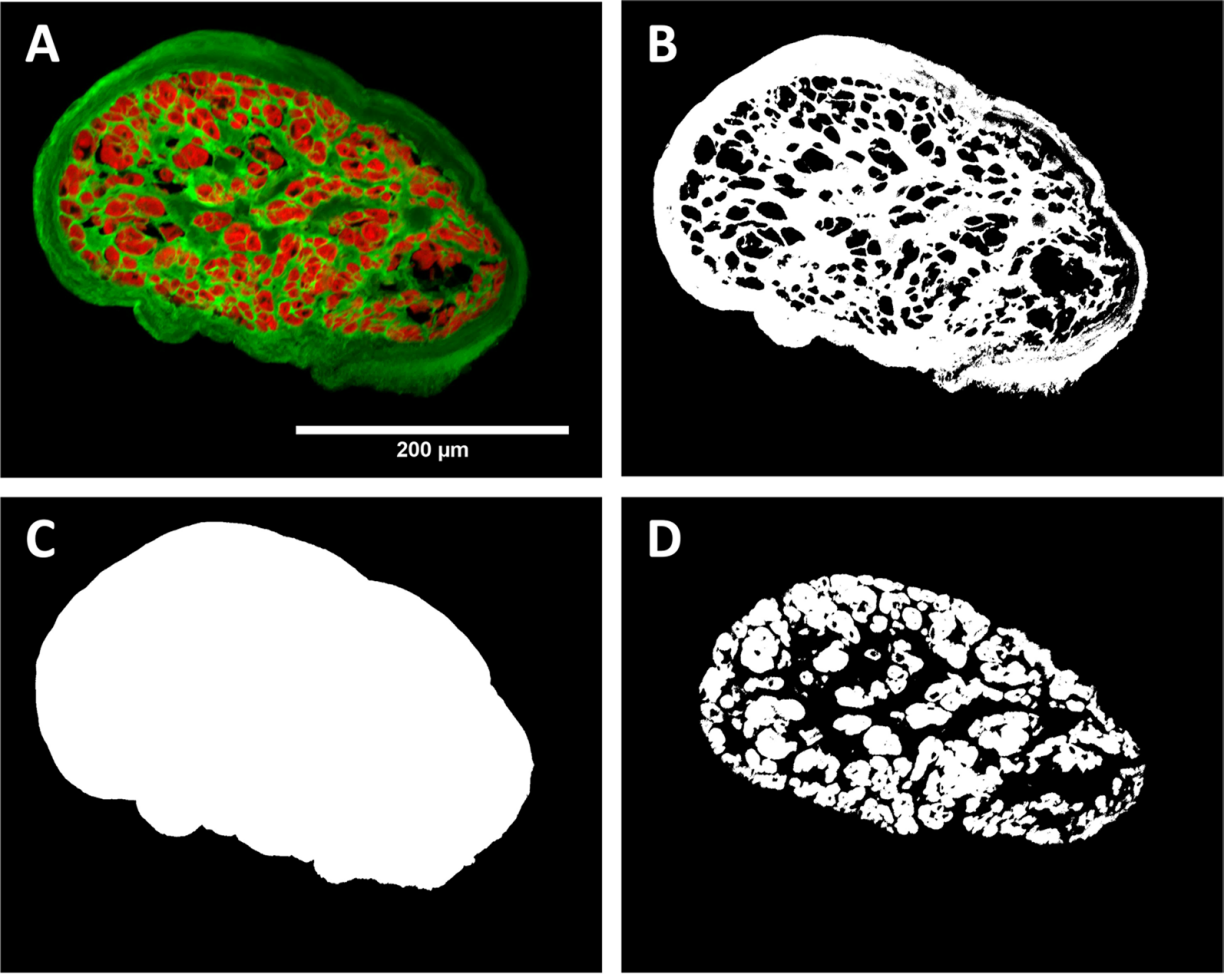
A sample confocal image showing the analysis performed in ImageJ. **A** trabecula cross-section with phalloidin (labelling f-actin) in red and WGA (labelling cell membrane and extracellular matrix) in green, **B** identified WGA area (green in panel A), **C** mask of the total cross-sectional area of the trabecula, **D** identified phalloidin area (red in panel A). The myofilament fraction for this section was 0.36

Images of the trabecula cross-sections were acquired with a Nikon C1 confocal microscope (20$\times$ objective) and EZ-C1 software (e.g. Fig. 1A) and processed semi-automatically in ImageJ.

WGA labelling (Fig. 1B) was used to select the trabecula perimeter and create a mask (Fig. 1C) whose pixels were counted to find the cross-sectional area of each section. The myofilament area in each section was similarly found by counting the pixels with phalloidin labelling above a certain threshold (Fig. 1D) and used to determine the fraction of the total area made up of myofilaments (phalloidin). This provides a reasonable proxy for the entire myocyte area. For each trabecula, the myofilament fraction and the total area of the cross-section were averaged across all sections which were successfully processed (at least two for each muscle). The fraction of myofilament content in each trabecula was compared between the non-diabetic and diabetic muscle groups by unpaired two-tailed t-test.

The measurement of trabecula cross-sectional area was used to normalise the measured forces for the final active and passive stresses presented in “Results” section. Additionally, the myofilament area was used to normalise the active stresses measured in the trabeculae which provides a measure of the contractile properties of the myofilaments themselves, rather than the entire muscle. This distinction was made as we expected these human muscles to contain varying amounts of extracellular matrix materials [16].

### Myofilament protein analysis (LC-SWATH-MS/MS)

Mass spectrometry analysis of the myofilament proteins was performed using the flash-frozen tissues from the RAA samples. These were acquired from a group of eight diabetic and six non-diabetic patient samples from which trabeculae used for the previously described experiments were obtained. Endocardial tissue pieces of around 5 mm $\times$ 5 mm $\times$ 2 mm were placed into 150 μL of lysis buffer containing 7 M urea, 2 M thiourea, 5 mM DTT in 50 mM ammonium bicarbonate and probe sonicated using a Qsonica Q125 ultrasonic processor (Qsonica LLC, CT, USA) for 4$\times$30 s at 30% amplitude. Samples were then centrifuged at 16,000 g for 5 min at 5 °C and an aliquot (50 μg of total protein) was taken from each sample and subsampled. Disulphide bonds were then reduced at 56 °C for 15 min in a temperature-controlled microwave (CEM, Mathews, NC, USA). Cysteines were alkylated with 15 mM iodoacetamide (IAM) in the dark for 40 min. 10 mM Cysteine was added to quench any residual IAM. The sample was then diluted 10-fold with 50 mM ammonium bicarbonate, and 1 μg of sequencing-grade modified porcine trypsin (Promega, Madison, WI, USA) added. Samples were incubated at 39 °C overnight in a temperature-controlled mini-incubator. The next day the digested samples were acidified with a small volume of 50 % formic acid, and then desalted on a 10 mg Oasis HLB SPE cartridge (Waters, MA, USA), eluting with 300 μL of 50% acetonitrile. The extract was then concentrated to approximately 25 μL in a vacuum centrifuge (Thermo Savant, Holbrook, NY, USA).

Sample extracts were analysed at 30-fold dilution with 0.1% formic acid and 8–10 μL injected (as appropriate based on signal intensity) onto a 0.3 mm $\times$ 10 mm trap column packed with 3 μm 300 Å C4 media (Dr Maisch, Ammerbuch-Entringen, Germany) and desalted for 3 min at 15 μL/min, before being separated with the following gradient at 6 μL/min using a Micro M5 UPLC system (AB SCIEX, Framingham, MA, USA): 0 min 1% B; 0.1min 5% B; 16 min 40%B; 17.5 min 98% B; 19.5 min 98% B; 20 min 1% B; 30 min 1% B, where A was 0.1% formic acid in water and B was 0.1% formic acid in acetonitrile.

The LC effluent was directed into a ZenoTOF 7600 Quadrupole-Time-of-Flight mass spectrometer (AB SCIEX) for SWATH acquisition comprised of 50 variable width isolation windows (with 1 Da overlap) covering a precursor mass range of 350–1100 m/z. The accumulation time was 50 ms for the initial TOF-MS scan, and 15 ms for each SWATH MS/MS scan (140–1600 m/z), giving a total cycle time of approximately 0.85 s. The mass spectrometer and UPLC system were under the control of the Sciex OS software package (AB SCIEX).

The resulting 14 MS/MS datasets were collectively searched against a Human protein database downloaded from Uniprot using DIA-NN 1.9.2 and output was filtered in DIA-NN quantification at 0.01 false discovery rate for precursors (peptide level) with no Heuristic protein inference, and Protein Inference set to Genes in order to distinguish between isoforms. Trypsin was set as a digestion enzyme allowing up to 1 mis-cleavages, the Iodoacetamide was set as global cysteine Alkylation modification, scan window was set to 10, MBR was enabled, Mass Accuracy was set to 25 ppm, MS 1 Accuracy to 15 ppm, charge 2–5, and fragment range 140–1800.

Of particular interest in this dataset were the relative proportions of alpha and beta heavy chain myosin isoforms in the tissue samples. Alpha myosin is the dominant isoform in human atrial tissue [17], but has been found at reduced proportions in certain disease states. For each patient sample, the unique abundances of MYH6 (alpha) and MYH7 (beta) were identified and the amount of alpha myosin was expressed relative to the total amount of both isoforms. This fraction of alpha myosin was compared between the non-diabetic and diabetic patient groups by unpaired two-tailed t-test.

## Results

### Force production

**Fig. 2:**
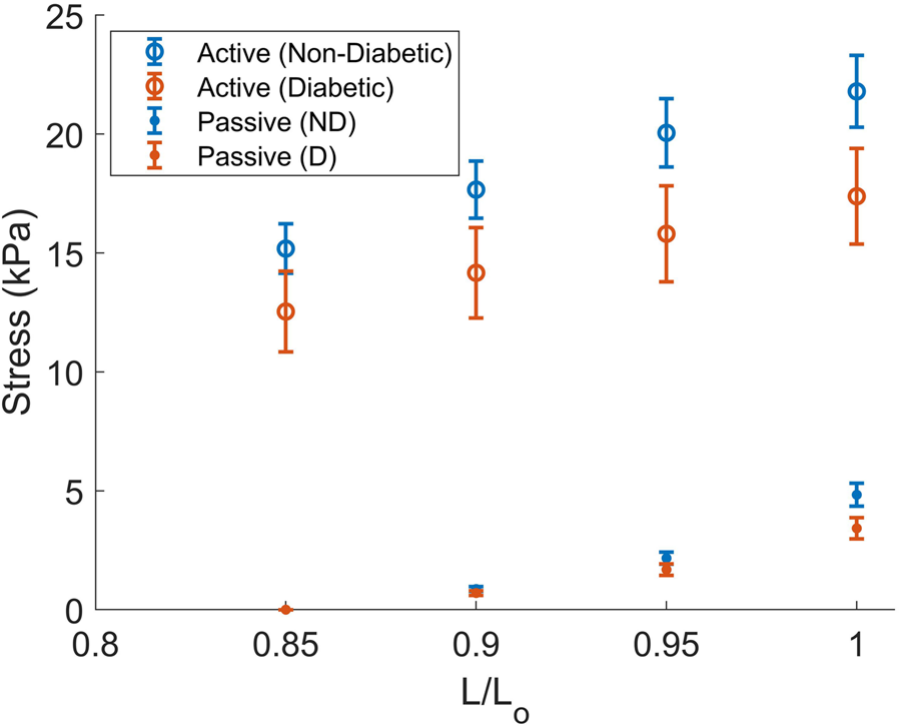
Active (unfilled circles) and passive (filled circles) steady-state stresses for the non-diabetic (blue) and diabetic (orange) trabeculae measured across different muscle lengths. $L/L_o$ represents fraction of the optimal length (2.2 μm sarcomere length). Active stress was found by subtracting the passive stress from the total stress measured in activating (pCa 4.5) solution. Diabetes had a significant effect on both active and passive stress across these lengths (two-way ANOVA, group effect)

The steady-state stresses measured during the initial mechanical protocol were higher among the trabeculae from non-diabetic patients than those from diabetic patients (Fig. 2).

The active stress developed in pCa 4.5 at optimal muscle length was 21.8 kPa ± 1.5 kPa for the non-diabetic trabeculae and 17.4 kPa ± 2.0 kPa for the diabetic trabeculae and the effect of diabetes on the active stress was significant over the four muscle lengths measured ($p<0.005$, two-way ANOVA group effect). There was also a significant effect of diabetes on the passive stresses over these four lengths ($p=0.047$, two-way ANOVA). At optimal length, a pairwise Tukey comparison showed that 4.8 kPa ± 0.5 kPa for the non-diabetic trabeculae was statistically higher than 3.4 kPa ± 0.4 kPa for the diabetic trabeculae ($p=0.008$).

**Fig. 3:**
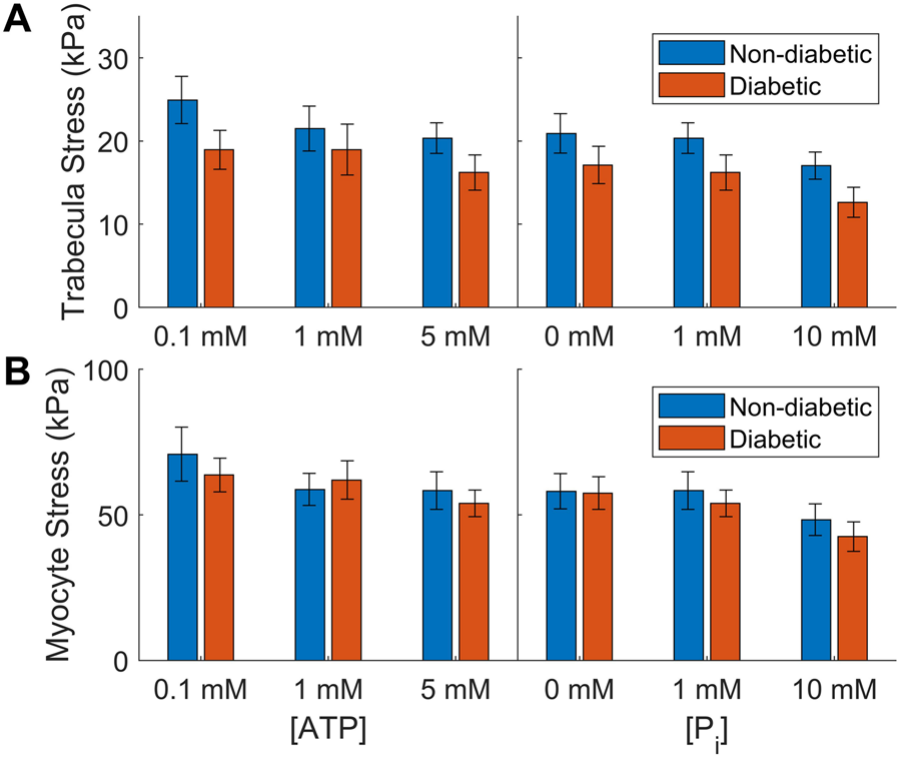
Active stresses developed at steady-state under different concentrations of ATP and P_i_ for non-diabetic (blue) and diabetic (orange) muscles. 5 mM ATP and 1 mM P_i_ both show the data measured under baseline conditions. Panel A shows the data measured where force is normalised to trabecula cross-sectional area. Here, the stress produced in the diabetic group is lower than the non-diabetic group across the five metabolite conditions ($p<0.01$, two-way ANOVA group effect). Panel B shows the same data normalised to the myofilament area, calculated as described in Section Trabecula structural imaging. Here, there is not a significant difference in the stresses produced by either group ($p=0.45$, two-way ANOVA)

The trend of higher active stress in the non-diabetic trabeculae continued across all five metabolic conditions studied in the experiments (Fig. 3A). Two-way ANOVA revealed a significant group effect on the active stress across the five conditions ($p<0.01$). On average, the stress produced by diabetic muscles was 20 % lower than that produced by the non-diabetic muscles.

To better understand the contractile properties at the individual myocyte level, stresses within the myocyte were estimated by normalising the force produced by the muscle to the myofilament area. This produced much larger steady-state stresses and reduced the difference in the stresses produced by the two groups (Fig. 3B). Unlike when the stresses were found from normalising to the entire trabecula cross-section, there was not a significant effect of the diabetic group across the active myocyte-level stresses measured under different metabolites ($p=0.45$, two-way ANOVA). Normalising passive stresses to the myofilament area also eliminated the significant effect of diabetes seen in Fig. 2 ($p=0.29$, two-way ANOVA, data not shown).

When looking at the effect of ATP and P_i_ separately, neither metabolite had a significant effect on the active stress ($p=0.35$ and $p=0.10$ two-way ANOVA metabolite condition effect, respectively). However, both the diabetic and non-diabetic groups showed typical trends of decreasing stress with higher concentrations of either ATP or P_i_, consistent with what we have previously measured in rat trabeculae under the same protocol [9]. While the two-way ANOVA did not reveal a significant interaction effect between disease group and metabolite condition, the sensitivity to P_i_ appears to be slightly higher in the diabetic group, with larger decreases in stress seen at 10 mM P_i_. These data also have a trend in the response to ATP that is different between the two groups. The non-diabetic muscles showed little difference in the stress produced between 1 mM ATP and 5 mM ATP, suggesting that the muscles are saturated at these higher ATP values. On the other hand, the diabetic trabeculae had a larger difference in stress here than between 0.1 mM ATP and 1 mM ATP. This suggests a different shape of the stress vs ATP curve in the diabetic trabeculae.

### Cross-bridge kinetics

**Fig. 4:**
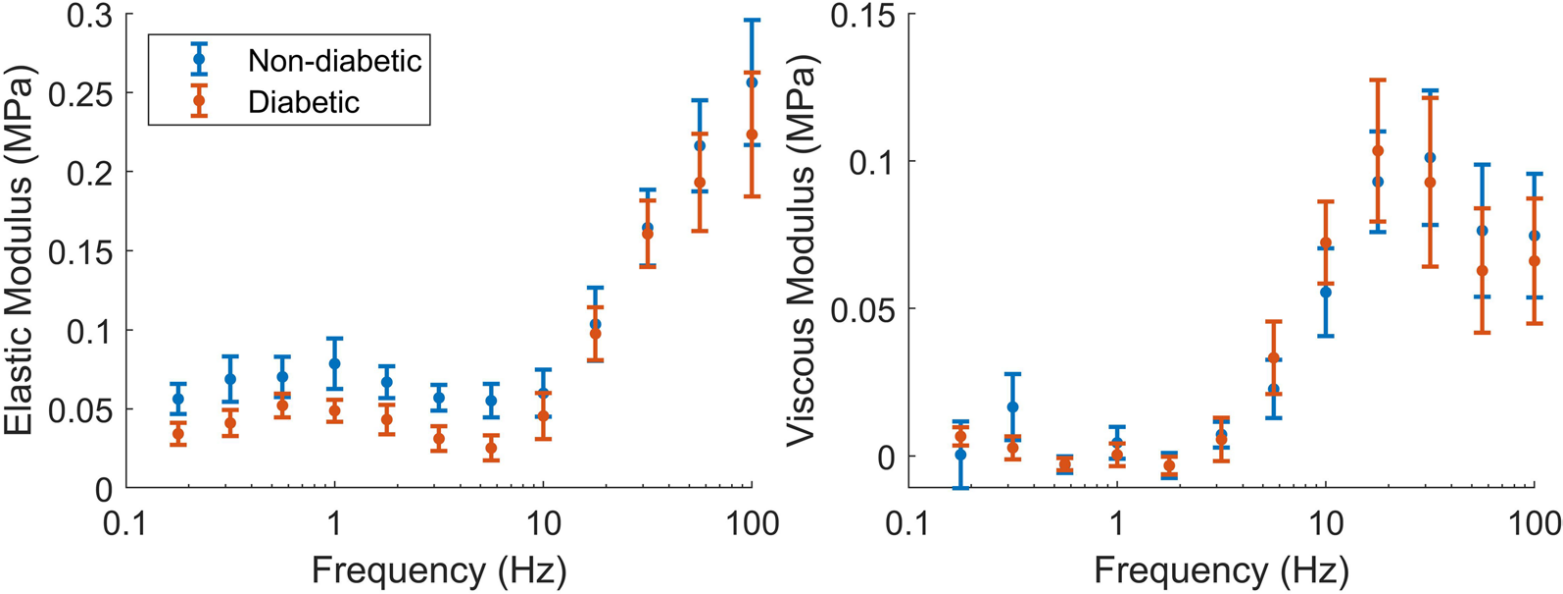
Active complex modulus at baseline metabolite conditions for non-diabetic and diabetic trabeculae. Across all 12 frequencies, the magnitude of the active complex modulus was lower in the diabetic group than the non-diabetic group ($p=0.03$, two-way ANOVA)

The differences in active stresses measured at steady state were also reflected in the stiffness measured in the active complex modulus (Fig. 4). Statistical analysis revealed that the magnitude of the complex modulus (dynamic modulus) was, on average, 16 % lower in the diabetic group compared to the non-diabetic group ($p=0.03$, two-way ANOVA group effect). As the elastic modulus contains most of the magnitude, the difference is most obvious in the left panel. There is also a subtle frequency shift to the left in the diabetic complex modulus. This was confirmed by fitting the 2-process exponential model (Eq. 1) to each complex modulus measurement, and confirming that the average rates of the B and C processes were both lower for the diabetic data (*b* = 6.1 Hz vs 7.1 Hz and *c* = 12.6 Hz vs 17.5 Hz).

The active complex modulus represents the resistance of the cycling cross-bridges to the external perturbation at different frequencies. A change in the magnitude of this stiffness reflects a change in either the number of attached cross-bridges in the muscle or a change in the stiffness of the average cross-bridge head. A shift to the left reflects a decrease in the frequencies of the underlying cross-bridge cycling rates.

**Fig. 5:**
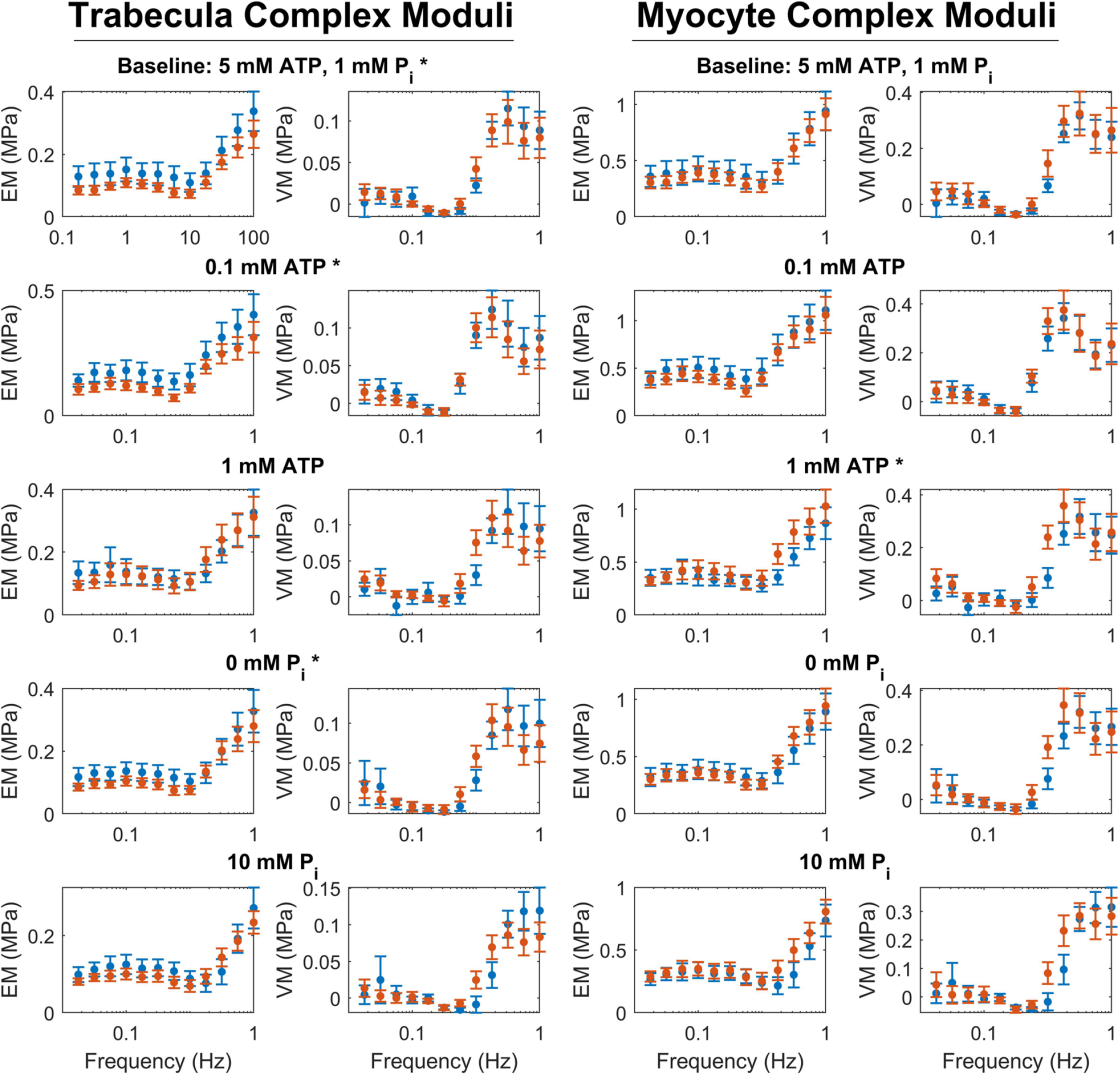
Effect of diabetes on the active complex moduli under different metabolite conditions, where stress is normalised to trabecula area (left panels) and to myofilament area (right panels). EM = elastic modulus, VM = viscous modulus. Symbol * marks metabolite conditions where diabetes had a significant effect on the dynamic modulus ($p<0.05$, two-way ANOVA)

Comparing the active complex moduli measured under each of the different metabolite conditions (Fig. 5) further demonstrates the key trends seen previously. Three-way ANOVA was performed on all the magnitudes of the trabecula complex moduli and revealed that both diasese group and metabolite condition had a significant effect on this stiffness metric ($p<0.001$ for both).

When considering each metabolite condition individually, all complex moduli magnitudes were different between the groups ($p<0.05$, two-way ANOVA), except for at 10 mM P_i_ ($p=0.12$) and at 1 mM ATP ($p=0.71$). The lack of difference at 10 mM P_i_ suggests lower sensitivity to P_i_ in the diabetic muscles, opposite to what was seen in Fig. 3, while the lack of difference at 1 mM ATP supports the abnormal ATP sensitivity that was suggested in Fig. 3. The magnitude of the non-diabetic modulus is clearly higher than that of the diabetic muscle at baseline metabolite conditions. As diabetic muscle is more sensitive to ATP at these concentrations, its modulus magnitude increases when ATP is lowered to 1 mM. There is little change in the non-diabetic modulus in this concentration range, so the two moduli are much more similar at 1 mM ATP.

Overall, the active complex modulus was more similar between the two groups when normalised to the myofilament area (Fig. 5 right panels). There was no significant difference between the non-diabetic and diabetic complex modulus magnitudes across a three-way ANOVA ($p=0.577$). There was, however, a significant difference between these values at 1 mM ATP, where the diabetic complex modulus has a higher magnitude than the non-diabetic measurement ($p=0.018$, two-way ANOVA). In addition, the three-way ANOVA model for the myofilament dynamic moduli had a significant interaction effect between diabetic group and metabolite condition ($p=0.045$), indicating that there is a difference in metabolite sensitivity between the two groups. This value also approached statistical significance for the trabecula dynamic moduli ($p=0.079$). When comparing the metabolite conditions with different ATP or P_i_ individually, the interaction effect was significant between diabetic group and ATP concentration ($p=0.029$), but not between diabetic group and P_i_ concentration ($p=0.313$).

**Fig. 6:**
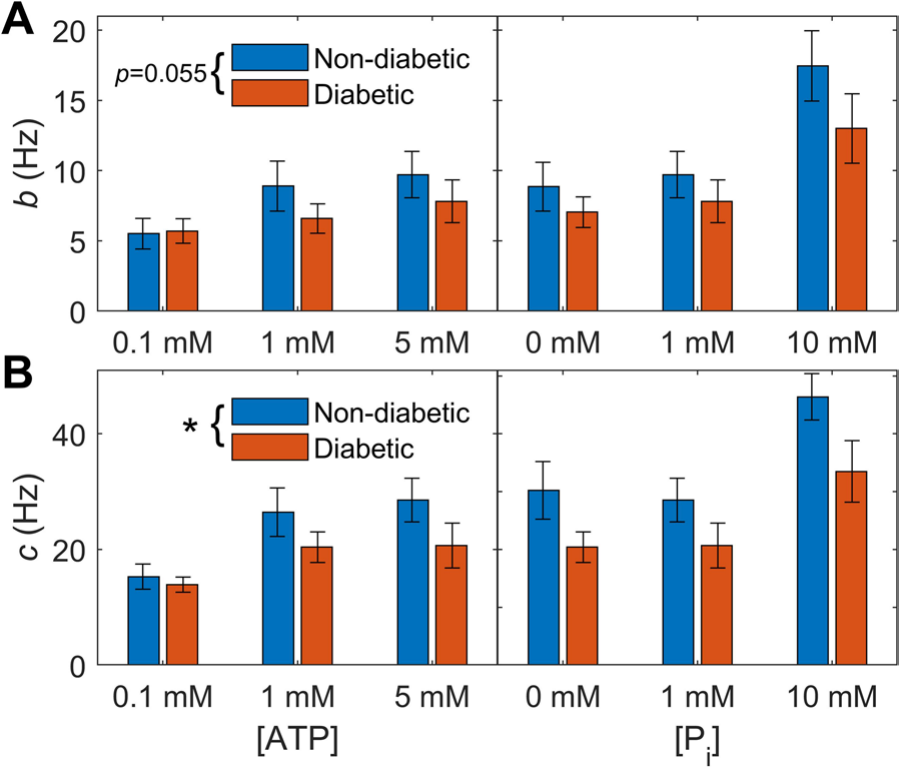
Average B and C process rates from a fitted exponential model (Eq. 1) across different ATP and P_i_ concentrations for diabetic and non-diabetic complex moduli. Across the five metabolite conditions, the rates of these processes were significantly lower in the diabetic muscles for the C process (panel B, $p<0.01$) and tended to be lower for the B process (panel A, $p=0.055$)

The leftward shift in complex moduli in the diabetic group is also visible at certain metabolite concentrations, most evident where the diabetic magnitudes are higher (e.g. at 1 mM ATP and 10 mM P_i_). The frequency behaviour was examined more closely by comparing the rates of the B and C processes of an exponential model fitted to these data (Fig. 6). The mean values of *b* and *c* tended to be higher in the non-diabetic muscles under almost all metabolite conditions, demonstrating the trend of a leftward shift in complex moduli in the diabetic group. This approached statistical significance for *b* ($p=0.055$, two-way ANOVA group effect) and was significant for *c* ($p<0.01$). On average, *b* was 22 % lower and *c* was 27 % lower in the diabetic muscles.

### Cardiac tissue composition

**Fig. 7:**
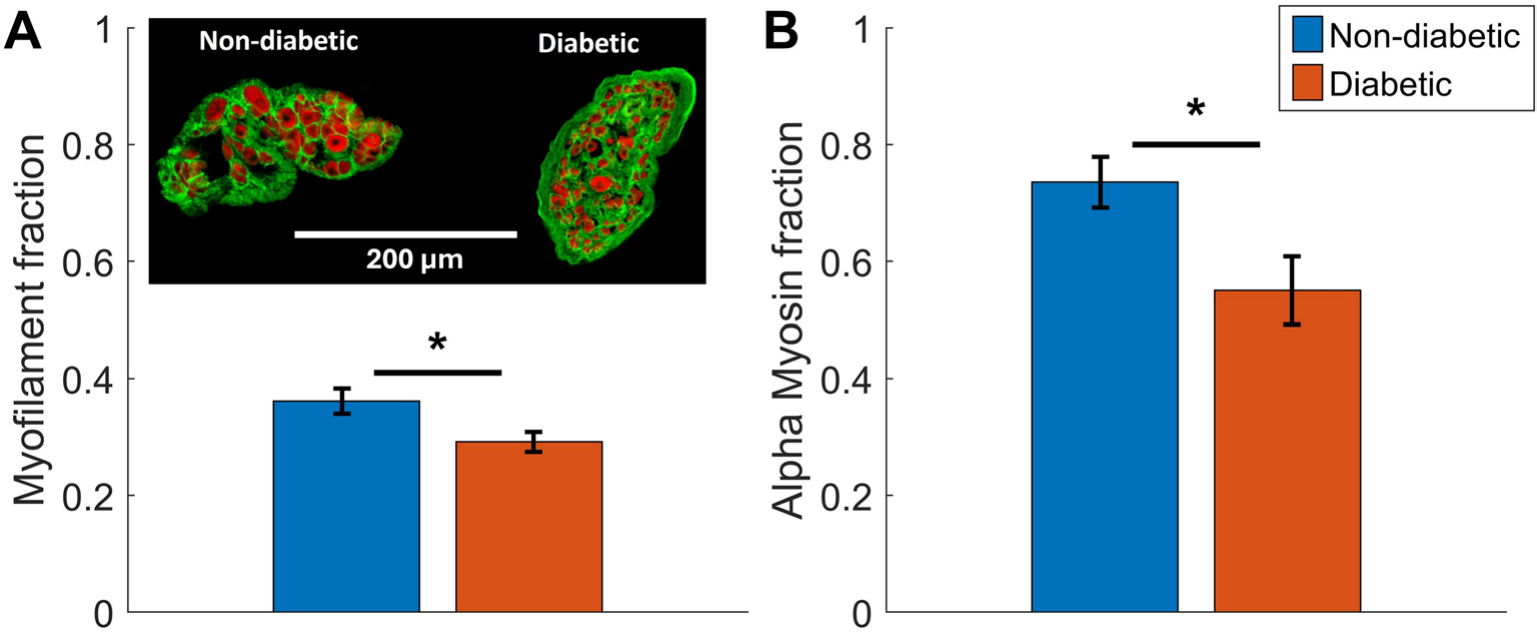
Structural studies on non-diabetic and diabetic atrial tissues. A: Comparison of myofilament fraction between trabeculae from non-diabetic and diabetic patients ($n=10$ for both groups). Myofilament fraction was found as the proportion of f-actin in the trabecula cross-section found via immunolabelling (see Fig. 1). Inset shows a representative section from each group (myofilament fraction: 0.35 vs 0.24). B: Comparison of alpha myosin isoform fraction between tissues for non-diabetic ($n=6$) and diabetic ($n=8$) patients. Alpha myosin fraction was found from the relative quantities of alpha and beta myosin isoforms measured using SWATH-MS. Symbol * indicates $p<0.05$ via t-test

To provide further insight into the results from functional measurements, we investigated the composition of the human atrial trabeculae. First, using immunohistochemistry and confocal imaging to explore the trabecula macrostructure, we measured a significant difference in the myofilament content between trabeculae from patients with and without diabetes (Fig. 7A). The non-diabetic trabeculae had an average myofilament area fraction of 0.36 ± 0.02, compared to a lower fraction of 0.29 ± 0.02 in the diabetic trabeculae ($p=0.02$, t-test). This reflects the myocyte density in the muscle cross-section and therefore demonstrates that there is a lower density of myocyte or myofilament content in the diabetic trabeculae. This conversely implies that there is a higher proportion of extracellular matrix material, collagen or fat content in the diabetic trabeculae. Note that there was no evidence that the average muscle cross-sectional area between the two groups was different ($p=0.81$, t-test).

Secondly, we used SWATH-MS mass spectrometry to explore the protein composition within the atrial tissues. From these data, we measured a significant difference in the fraction of myosin heavy chain proteins that were in the alpha isoform rather than the beta isoform (Fig. 7B). The non-diabetic tissues had a higher average alpha myosin fraction of 0.74 ± 0.04 compared to the diabetic tissues with a fraction of 0.55 ± 0.06 ($p=0.034$, t-test). This conversely reflects a higher fraction of the beta myosin isoform in the diabetic tissues. Beta is the slower myosin heavy chain isoform, so this result is consistent with the slower cross-bridge cycling rates measured in the diabetic muscles.

## Discussion

Our suite of experimental methods focussed on capturing cross-bridge function revealed several differences between permeabilised cardiac trabeculae from patients with diabetes and those without. From the cohort of right atrial appendage trabeculae examined in this study, we have found that diabetic muscles had significantly lower active stresses, passive stresses, complex modulus magnitudes and lower characteristic frequencies in the active complex modulus. From our structural studies, we also found that the diabetic muscles had a lower myofilament density and lower proportions of the alpha myosin isoform. Both groups of muscles exhibited typical responses to ATP and P_i_ concentration changes, but with slightly altered sensitivity. The functional differences we found were consistent with the tissue structural changes and involve tissue or cell-level phenomena which reflect clinical changes often seen in diabetic heart disease.

### Stress production is lower in diabetic tissues

The reduced active stress we measured in diabetic trabeculae (Figs. 2 and 3) is in agreement with other experimental studies performed on human cardiac tissue. Jones et al. [15] studied intact right atrial trabeculae from the same cohort of CABG patients and found a reduced twitch amplitude for diabetic samples. While it was postulated that this was primarily caused by a reduction in Ca^2+^ transient amplitude, reproducing this effect in maximally-activated permeabilised muscles in this study supports the hypothesis that there is also an impairment in the contractile proteins themselves. In a study by Jweied et al. [18], where permeabilised myocytes were acquired from left-ventricular biopsies of patients undergoing CABG surgery, they also found that myocytes from diabetic patients produced less force at saturating Ca^2+^ concentrations. However, these and our findings are in contradiction with those of Fukagawa et al. [19], who studied myocardial strips from the left ventricle and found no significant differences in the maximum stress developed. Fukagawa et al. [19] performed their experiments at 35 °C, as opposed to the 15 °C used by Jweied et al. [18], so they may have more relevance to the results we found at body temperatures.

In mechanistic models of contraction, the main determinants of steady-state stress are the proportion of strongly-bound cross-bridges at steady-state and the stiffness coefficient of the cross-bridges [9]. These variables also scale the complex modulus magnitude, so it is unsurprising that we found the effects of diabetes and metabolite conditions on active stress were similar for active complex moduli (Figs. 4 and 5). The Fukagawa et al. [19] study also looked at complex modulus measurements but did not find any differences between the magnitude of the total complex moduli measured from the two disease groups. However, as they did not report active complex moduli, their measurements do not directly reflect the cross-bridge properties of the muscle. They did report passive dynamic moduli, which tended to be higher in the diabetic males, suggesting that the active dynamic modulus may indeed be lower for this group, in line with our data in Fig. 5.

Our results of lower active stress and stiffness provide evidence of contractile dysfunction in the diabetic muscles studied. Although cardiac cells in vivo are not maximally activated, cross-bridges with a lower stiffness coefficient or fewer cross-bridges in their force-generating state will reduce the contraction force produced during a normal cardiac cycle and could lead to systolic impairment. Systolic dysfunction is considered a feature of later-stage diabetic cardiomyopathy [2] or may only be present to a subclinical degree [3], which may explain why similar findings of contractile function are not seen consistently in literature. In our cohort, none of the patients had heart failure, and while there was not a significant difference, the non-diabetic group had slightly worse systolic function than the diabetic group. These data suggest that signs of contractile dysfunction at the tissue level may precede more obvious clinical symptoms of impaired organ function in diabetes.

### Impact of myofilament fraction on cross-bridge behaviour

The measurements of trabecula macrostructure offer a compelling explanation for the reduced stresses and stiffnesses measured in the diabetic muscles. Based on prior evidence that human cardiac trabeculae exhibit a large range of myocyte densities [16], every trabecula that was studied was also immunolabelled with phalloidin and analysed to determine its myofilament content. This allowed for comparison of the myofilament fraction in trabeculae across the two groups, and to quantify the stress produced by the myofilaments themselves.

The first finding from this structural analysis is that the trabeculae studied did contain quite low amounts of myofilament, approximately 33 % of the muscle cross-section on average. This is lower than what Munro et al. [16] found in human ventricular trabeculae, but similar to what Jones et al. [15] found in human right atrial samples. In line with the trend seen by Jones et al. [15], we found that the myofilament density was lower in the trabeculae from diabetic patients (Fig. 7A). Figure 3B and the right panels of Fig. 5 showed that there are no longer significant differences in active stresses and dynamic moduli when the force is normalised to myofilament area. Therefore, we can conclude that the lower stress and stiffness measurements in diabetic muscles are primarily due to a lower number of myofilaments (or cross-bridges) contributing to the activation of the muscle. Jones et al. [15] still saw a difference in peak stress when normalising to myofilament area, suggesting that the difference in intracellular Ca^2+^ was also an important factor in their results from intact muscles.

Since the myofilament fraction measured was treated as a proxy for myocyte content, normalising the force to myofilament area should produce outputs closer to those measured in individual myocytes. The magnitudes of these stresses are around 60 kPa at baseline conditions, which agrees well with the maximal steady-state stresses reported by Land et al. [20] for permeabilised myocytes held at body temperature and 2.2 um sarcomere length.

A lower proportion of myofilament in the diabetic group is not a surprising finding, given the well-established association between diabetes and cardiac fibrosis [21]. While immunolabelling did not include detailed analysis of the non-myofilament regions of the trabeculae, we found that the WGA-labelled area was higher in the diabetic muscles. As WGA has been shown to label fibrosis [22], this higher fraction of WGA likely indicates more fibrosis in the diabetic trabeculae, as would be expected with a lower proportion of myocytes. There may be a combination of mechanisms contributing to fibrosis in the studied cardiac trabeculae, including increased fibroblast activation and expanded extracellular matrix deposition, or replacement of myocytes with fibrotic tissue following cell death [21, 23, 24].

### Slower detachment kinetics in diabetic trabeculae

While the differences in magnitude of the complex moduli between the two groups are diminished by accounting for the myofilament fraction, the characteristic frequencies cannot be explained by this finding. We found lower frequencies for the exponential processes in the active complex modulus data from the diabetic muscles (Fig. 6). This effect is evident in our plots of active complex modulus data by a leftward shift in the diabetic group, when normalised to either trabecula area or myofilament area (Fig. 5). From the classical understanding of muscle complex moduli measurements, a leftward shift in frequencies reflects slower cross-bridge kinetics. Our previous work analysing biophysical cross-bridge models demonstrated that a reduction in cross-bridge detachment rates specifically can cause a leftward shift in frequency [9]. Slower cross-bridge detachment rates will directly slow down relaxation in intact muscle and will contribute to diastolic dysfunction if the muscle does not have time to relax fully between beats. This would be particularly likely to occur at the higher heart rates experienced by diabetic patients [25], or during exercise.

A leftward shift in the complex modulus was seen in diabetic samples by Fukagawa et al. [19], but only when comparing the female groups. We would expect our results to align more closely with their results from males, since the majority of our cohort were mostly male patients (Table 1), but Fukagawa et al. [19] saw the opposite effect in their male groups. While these existing complex modulus measurements do not consistently support our findings, other metrics of reduced cross-bridge cycling rates, such as reduced shortening velocity and prolonged twitch duration, have been previously observed in a range of intact diabetic tissue experiments [26–29].

The faster of the two common cardiac myosin isoforms (alpha) has previously been found to be present at lower levels in diabetic rat hearts [30, 31], as well as in the ventricles of humans with heart failure [32]. We therefore hypothesised that this shift toward the slower (beta) isoform may underlie the slower cross-bridge cycling kinetics that we measured in this study. To our knowledge, this measurement has not been made in diabetic human tissues previously. SWATH-MS mass spectrometry allowed us to differentiate the relative proportion of alpha and beta myosin in the atrial tissues of patients in the study and revealed that there was a shift toward beta myosin among the diabetic group (Fig. 7B). This provides further evidence of slower cross-bridge cycling in the diabetic muscles and provides a clear mechanism underlying this change.

### Differences in metabolite sensitivity of diabetic trabeculae

All of the active muscle measurements were performed under three different concentrations of ATP and P_i_ to assess the metabolite-dependent behaviour. While both groups seemed to respond typically to changes in metabolite, there was an interaction between diabetic groups and metabolite condition in the active dynamic modulus, significant when normalised to myofilament area (Fig. 5, right panels). This indicates that there is a difference in the sensitivity to metabolites in the diabetic muscles. The two groups appear to exhibit slightly different sensitivity to both ATP and P_i_, although the interaction effect was only significant for ATP when considered individually.

The non-diabetic muscles have similar stress and modulus values at 1 mM and 5 mM ATP. A saturated response is expected across these ranges, given that typical $k_\textrm{d}$ values for ATP are well below 1 mM [33, 34]. However, the diabetic muscles do not seem to saturate at this level, instead showing similar differences in complex moduli across all ATP levels. As patients with type 2 diabetes have reduced availability of ATP [5], a lack of saturation around resting levels of ATP means that the function of diabetic cross-bridges may be altered in vivo due to a small decrease in ATP concentration, unlike what is typically seen [34]. Lowered ATP could have a compensatory response in increasing the stress production, but would also cause slowing of the cross-bridge cycling and further impair relaxation in the diabetic muscles.

While the steady-state stresses generated in the two groups appeared to show higher sensitivity of diabetic muscles to P_i_ concentration, non-diabetic muscles showed a larger change in complex modulus magnitude under 10 mM P_i_ concentrations. As diabetic muscles are more likely to experience elevated P_i_ levels [6], this could represent a compensatory mechanism in the diabetic muscles to maintain their stiffness under these conditions.

While three concentrations of ATP and P_i_ were sufficient to characterise cross-bridge sensitivity to metabolites in a modelling study [9], further study may be required here to better identify any subtle changes in metabolite sensitivity, particularly the effect of P_i_.

### Passive stress in diabetic trabeculae does not indicate diastolic dysfunction

A surprising result of this study was the reduction in passive stress seen in the diabetic muscles. Diastolic dysfunction is a typical early symptom of diabetic cardiomyopathy [2] and our patient data showed significantly higher E/e′ ratios in the diabetic group (Table 1). While the mean values are below the clinical threshold, this difference indicates that the diabetic patients may be progressing toward diastolic dysfunction at the organ level. As discussed previously, the higher proportion of WGA-labelled area and lower proportion of myofilaments in the diabetic trabeculae provide strong evidence that there is increased fibrosis in the diabetic tissues, which is generally considered to drive diastolic dysfunction in diabetic cardiomyopathy. Based on these factors, we expected to see raised levels of passive stress in the diabetic group. However, Jones et al. [15] saw elevated diastolic Ca^2+^ in their diabetic trabeculae despite no difference in the diastolic force between the two groups, suggesting the passive stress was likely lower in the diabetic group. In conjunction with these similar results, our findings thus suggest that diastolic dysfunction at the tissue level, at this stage of the disease, is related to Ca^2+^ handling and the reduced relaxation kinetics, rather than the passive tissue properties themselves. Since there is no guarantee that the heart achieves a fully relaxed state during diastole, it is difficult to ascertain how much the true passive properties are impairing relaxation in vivo.

The reduced passive stress measured in the diabetic muscles is likely due to the lower myofilament fraction. As with the active stresses, normalising the passive stress to the myofilament area, instead of the trabecula area, eliminates the significant differences seen in Fig. 2 (data not shown). This indicates that passive forces are similar between the two groups at the myocyte level and suggests that lower passive stress in the permeabilised diabetic trabeculae is due to less titin and other intracellular structures arising from a loss of myocytes.

### Limitations

An important limitation to consider in this study is the applicability of our findings in atrial tissue to ventricular tissue where diabetic cardiomyopathy primarily manifests. Atrial samples containing trabeculae are more readily available from patients, and we expect that diabetes affects myocytes similarly in all chambers of the heart. However, inherent differences between atrial and ventricular tissues may limit how well certain findings reflect the likely response to diabetes in the ventricles. Because the atria contract more rapidly than the ventricles, human atria typically have a higher fraction of alpha myosin than human ventricles [17, 35]. Despite this, human ventricular tissues have previously been found to exhibit significant reductions of alpha-myosin fraction in heart failure, often reducing to undetectable levels [32]. However, as the ventricles have less than 10% alpha myosin to begin with, we would expect that an isoform shift in the ventricles may not result in as pronounced a shift in cross-bridge kinetics as was measured in atrial tissue in our study.

The use of atrial trabeculae, rather than ventricular samples, may also explain why signs of diastolic dysfunction were not evident in the passive properties we measured. However, Fukagawa et al. [19] performed their experiments on human ventricular myocardial strips and found no difference in the passive stiffness between the disease groups either. Since we used similar permeabilisation protocols to this group, this raises the question of whether permeabilisation considerably alters the passive properties of tissue. A comparative study performed by Saeki et al. [36] showed no evidence of any difference between passive complex moduli measured in intact and permeabilised trabeculae from healthy ferrets. However, the possible effects of permeablisation on human and diabetic trabeculae cannot be ruled out. An alternative explanation for the absence of elevated passive stresses in diabetic trabeculae could be that the contribution of passive muscle properties to diastolic dysfunction observed in vivo may become apparent only at the organ level or within the ventricles. This hypothesis is supported by the clinical evidence of left ventricle remodelling in patients with diabetic cardiomyopathy [3].

We also note a potential limitation relating to the gender distributions in the non-diabetic and diabetic patient groups, specifically, a greater proportion of female patients in the diabetic cohort. While sex differences are prevalent in all aspects of biology, we do not expect that the different group compositions materially affected the findings of our study. Rat trabeculae studied under a comprehensive suite of mechanical metrics did not differ between male and female rats [37] and myosin isoform ratios in healthy and failing hearts were also found to be independent of sex [35]. Studies that do find an effect of sex on cardiac metrics relevant to our study tend to show that female hearts contract faster [19, 38], which would oppose the effect we saw in the diabetic group. Given that the diabetic cohort had a higher proportion of female patients, this strengthens our finding that diabetes slows cross-bridge cycling.

Finally, a limitation in analysing patient tissue data is the inherent variability of the patients themselves and potentially their medical history, even when the same surgery is performed. In our patient cohort, the only statistical differences in clinical characteristics between the groups were in HbA1c levels and E/e’ ratio. The former should always be higher in a diabetic group and the latter is not likely to have affected the findings of this experimental study. Despite any potential variation in medical history among the patients in this cohort, we were still able to measure and identify key differences between the mechanical function of heart tissues from diabetic and non-diabetic patients.

## Conclusions

In this study, the mechanical properties of 10 atrial trabeculae from patients with type 2 diabetes and 10 from patients without diabetes were measured and explored. We found evidence of differences at the cellular and tissue levels, which may manifest as typical clinical symptoms of diabetic cardiomyopathy.

The first finding was that trabeculae from patients with diabetes produced less active stress and exhibited lower cross-bridge stiffness. Based on the structural imaging, the difference in cross-bridge mechanics in these muscles originated from a lower density of myofilaments within the trabeculae. This would result in fewer cross-bridges being available for a given muscle cross-section and could contribute to contractile dysfunction at the organ level.

The second finding was slower cross-bridge kinetics evident in diabetic complex moduli. Measurement of the relative proportion of different myosin isoforms demonstrated that this difference was due to a shift towards the slower beta isoform of myosin. Slower cross-bridge kinetics align with the common symptom of diastolic dysfunction in diabetic cardiac muscle, as they impair relaxation, especially if paired with higher diastolic Ca^2+^ or higher heart rate. Although the diabetic trabeculae had lower myocyte content and potentially a higher fibrotic content, we measured lower passive stresses in these muscles, suggesting that passive mechanical properties may only be impaired at the organ level in diabetic cardiomyopathy.

As mitochondrial dysfunction plays a role in diabetic cardiomyopathy, we also assessed the sensitivity of the trabeculae to ATP and P_i_. The diabetic muscles presented with slightly different responses to metabolite concentration changes compared to non-diabetic trabeculae. The diabetic muscles had higher sensitivity to ATP around physiological concentrations and trends toward lower P_i_ which may further exacerbate the differences under the metabolic conditions diabetic cells experience in vivo.

## Data Availability

Averaged data presented in this study that are suitable for parameterising a metabolite-sensitive cross-bridge model (as in [9]) are available to download on figshare at https://doi.org/10.6084/m9.figshare.28180337. This includes data from both diabetic and non-diabetic groups. This repository also includes a complete MATLAB database of the raw data contributing to the results presented below. The authors may be able to provide these data in alternative forms upon reasonable request.
